# Early Developmental Exposure to Triclosan Impacts Fecal Microbial Populations, IgA and Functional Activities of the Rat Microbiome

**DOI:** 10.3390/jox14010012

**Published:** 2024-02-01

**Authors:** Mohamed Lahiani, Kuppan Gokulan, Vicki Sutherland, Helen C. Cunny, Carl E. Cerniglia, Sangeeta Khare

**Affiliations:** 1 Division of Microbiology, National Center for Toxicological Research, U.S. Food and Drug Administration, Jefferson, AR 72079, USA; hassen86us@gmail.com (M.L.); kuppan.gokulan@fda.hhs.gov (K.G.); 2 National Institute of Environmental Health Sciences, National Institutes of Health, Durham, NC 27709, USA; vicki.sutherland@nih.gov (V.S.); cunnyh@niehs.nih.gov (H.C.C.)

**Keywords:** antimicrobial, endocrine disrupting chemical, fecal IgA, gastrointestinal tract, microbiome, oral exposure, perinatal exposure, postnatal exposure, risk assessment, triclosan, toxicity, xenobiotic

## Abstract

Triclosan (TCS), a broad-spectrum antibacterial chemical, is detected in human urine, breast milk, amniotic fluid, and feces; however, little is known about its impact on the intestinal microbiome and host mucosal immunity during pregnancy and early development. Pregnant female rats were orally gavaged with TCS from gestation day (GD) 6 to postpartum (PP) day 28. Offspring were administered TCS from postnatal day (PND) 12 to 28. Studies were conducted to assess changes in the intestinal microbial population (16S-rRNA sequencing) and functional analysis of microbial genes in animals exposed to TCS during pregnancy (GD18), and at PP7, PP28 and PND28. Microbial abundance was compared with the amounts of TCS excreted in feces and IgA levels in feces. The results reveal that TCS decreases the abundance of *Bacteroidetes* and *Firmicutes* with a significant increase in *Proteobacteria*. At PND28, total Operational Taxonomic Units (OTUs) were higher in females and showed correlation with the levels of TCS and unbound IgA in feces. The significant increase in *Proteobacteria* in all TCS-treated rats along with the increased abundance in OTUs that belong to pathogenic bacterial communities could serve as a signature of TCS-induced dysbiosis. In conclusion, TCS can perturb the microbiome, the functional activities of the microbiome, and activate mucosal immunity during pregnancy and early development.

## 1. Introduction

Triclosan (TCS) is a synthetic broad-spectrum antimicrobial used since the early 1960s. Due to its positive effect, TCS has been incorporated into over 2000 health care products, toys, clothes and household consumer products [1]. Some examples of these include surgical suture materials, deodorants, shampoos, soaps, toothpastes, antiseptic creams, plastics, functional clothes, and many other products. From 1977 to 2011, TCS production increased from 0.5 million pounds/year to 14 million pounds/year [2]. In consumer products, the concentration of TCS ranges from 3.5 to 17 mM [3]. Studies have shown that human skin and oral mucosa are capable of absorbing TCS, which results in the detection of TCS in various tissues and body fluids [4,5]. Consumer and commercial product wastes are known to contaminate the environment and water sources and the presence of TCS in these materials could have an adverse impact on health [6,7]. Continuous exposure to TCS through various consumer products, as well as the deposition of TCS in river water and soil, leads to the emergence of antimicrobial-resistant bacteria [8,9]. TCS-resistant bacteria also showed resistance to other antimicrobial agents [10]. In 2017, the U.S. Food and Drug Administration (FDA) banned the use of TCS in over-the-counter antiseptic products [1]. However, TCS remains approved for use in other applications such as toothpastes, skin antiseptics, dental materials and implants [11,12]. TCS is found in urine [13], in pregnant women and their newborns [14,15,16] and in 75% of the general U.S. population [2,17,18]. The Environmental Protection Agency has classified TCS as a pesticide [19].

The presence of TCS as an additive in many consumer products resulted in direct contact with the microbiome present on the skin, oral cavity and in the gastrointestinal system. Despite the increasing interest in the impact of TCS on the microbiome, conflicting results exist on the potential adverse effects of this additive. TCS at low concentrations causes a bacteriostatic effect by inhibiting bacterial fatty acid synthesis [20]. In addition, low concentrations of TCS cause colonic inflammation and colon cancer and alter intestinal commensal bacteria in an azoxymethane/DSS–induced colon cancer model in mice [21]. At higher concentrations, TCS can be bactericidal [22]. In a 10-year study, Dhillon et al. found that TCS exposure did not lead to a change in methicillin-resistant *Staphylococcus aureus* or *Pseudomonas aeruginosa* tolerance to antibiotics [23]. On the other hand, Wesgate et al. found that after a low level (0.0004%) of TCS exposure, *S. aureus* and *E. coli* were resistant to ampicillin and/or ciprofloxacin [24]. Adult zebrafish exposed to TCS for 7 days displayed a significant modification in the intestinal bacterial community structure, interaction networks, and an increase in TCS resistance [25]. The alteration of the gut microbiome was also observed in fathead minnows exposed to environmentally relevant low levels (100 ng/L) of TCS [26]. However, the study also showed that this effect was reversible and the gut microbiome can recover after a short-term perturbation [26]. Many of the conflicting results are due to differences in study designs (e.g., dosing, time and route of exposure and/or the model used in the study). Moreover, current studies on the impact of TCS on the microbiome are inconclusive and unclear.

To elucidate the impact of TCS on the human gut microbiome, a study by Poole et al. used a control crossover strategy that allowed the use of each person as his/her own control. The study found no significant effect due to the exposure of household and personal care products containing TCS to the microbiome present in the gut or oral cavity [27]. However, these studies were not designed to determine whether prenatal, perinatal or postnatal TCS exposure can have long-lasting impacts on the microbiome. A study by Hu et al. found that adolescent rats were more prone to microbiota perturbation than adult rats after TCS exposure [28]. Another study showed that TCS is readily absorbed in the body, and its elimination via urine or feces is gender- as well as age-dependent [29]. This study showed that the serum TCS concentration were highest in the group of 31–45-year-old males and females; however, the concentrations were higher in males as compared to females.

Furthermore, there are emerging reports showing the association between TCS and other household disinfectants on human body mass index (BMI) [30,31]. The gut microbiome was shown to be a key player in childhood obesity with possible impacts on neurological and immune development [31,32,33]. The gut microbiome is influenced by components of the innate and adaptive immune system [34,35,36]. Immunoglobulin A (IgA) is the primary antibody secreted in the gut and can shape the composition of the microbiome [34]. Moreover, currently, there are no data on the effect of TCS on secretory IgA in feces.

Given that there are many gaps in our understanding of the impact of TCS on the microbiome and since many factors (age, gender, dose, exposure timing) play a role in the microbiome response, we examined how oral exposure to TCS affects the microbiome during pregnancy and early development in the rat.

**Figure 1:**
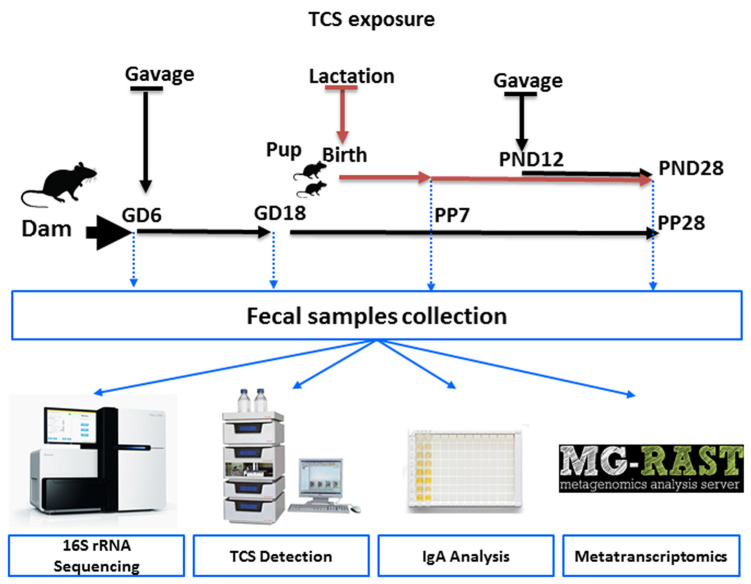
Schematic diagram of the study design. Pups were orally gavaged from postnatal day (PND)12 through PND28 at the same dose (100 or 500 mg/kg/day) as that of their dam.

The aim of this study was to evaluate the effect of TCS on intestinal commensal bacteria during pregnancy as well as during early development. Despite the ban on TCS in several daily-use products, this study provides clues whether the usage of TCS during pregnancy and lactation could cause any effects on the microbial population and functions in the progeny. TCS was administered via oral gavage as indicated in the schematic diagram (Figure 1) starting on Gestation Day 6 (GD6) to the pregnant dam and continued through birth and lactation until weaning. The offspring were directly dosed via oral gavage from postnatal day (PND) 12 to 28. In this study, our main objectives were: (i) to evaluate if there were any differences in the intestinal microbial community structure upon exposure to TCS during GD18 and lactation (PP7 and PP28), (ii) to identify the abundances of intestinal microbial bacterial species and functional contents encoded in the genomes of intestinal bacteria in pups on PND28 during TCS exposure, (iii) to examine if there was any correlation between excreted TCS in feces and the intestinal microbiome, (iv) to examine if there was any correlation between IgA and excreted TCS in feces, and v) to evaluate if there were possible dose effects in the male and female pup intestinal microbiome and functional activity after exposure to TCS.

## 2. Material and Methods

### 2.1. Animal Housing and Diet

All animals [pregnant female Sprague Dawley rats (Hsd:SD) and their male and female pups] were housed in the animal facility at Southern Research, Birmingham, AL, an independent scientific contract research organization. The Southern Research Institutional Animal Care and Use Committee reviewed the protocol and approved it. The IACUC number for this protocol is 17-01-005B. Animals were housed in polycarbonate cages with irradiated hardwood bedding chips (Sani Chips^®^; P.J. Murphy Forest Products Corp.; Montville, NJ, USA). Animals were fed irradiated NIH-07 pellets or wafers (Zeigler Bros., Gardners, PA, USA) and provided municipal water ad libitum; both water and feed were analyzed for known contaminants that could interfere with or affect the outcome of the study and none were found. Dams were individually housed, except during the lactation period when they were housed with their litters. Offspring remained with their respective dams until PND 28.

### 2.2. TCS Dose Selection and Exposure to Animals

The no-observed adverse effect level (NOAEL) dose for the safety and tolerability of TCS in various organ/cellular components varies considerably [37,38]. The developmental toxicity NOAEL for subcutaneous dosing is 100 mg/kg/day TCS [38]. However, primary exposure to TCS has been via the oral route. A perinatal toxicity study evaluating oral exposure at doses of 0, 100, 250, 500 or 1000 mg/kg/day recorded decreases in body weight gain at 250 mg/kg and above during treatment, with no evidence of developmental toxicity (unpublished data from a study conducted at the National Toxicology Program/NIEHS). Thus, for this study, the lowest dose (100 mg/kg/day) of TCS was selected based on the NOAEL for developmental toxicity in the perinatal study. Furthermore, the two higher doses were chosen for this study (500 and 1000 mg/kg/day) to assess the threshold for dose response to xenobiotics [39].

TCS was administered via oral gavage to time-mated female Sprague Dawley rats (Hsd:SD) starting on gestational day 6 (GD6) and continued through gestation and lactation (NTP study MOG03039). There were 6 groups in this study. Group 1: TCS 0 mg/kg/day dosed with 5 mL/kg corn oil (CO5); Group 2: TCS 0 mg/kg/day dosed with 2 mL/kg corn oil (CO2); Group 3: TCS at 100 mg/kg/day (TCS100); Group 4: TCS at 500 mg/kg/day (TCS500); Group 5: 1000 mg/kg/day (TCS1000); Group 6: untreated (C). The 1000 mg/kg treatment group was excluded after the first time point due to behavioral changes. All groups were dosed with a vehicle volume of 5 mL/kg corn oil except for group 2 which received 2 mL/kg corn oil and group 6 which was not dosed. Pups were orally gavaged from PND12 through PND28 at the same dose as their dam. The numbers of animals in each experimental group were as follows: GD6-C = 4, GD18-CO5 = 3; GD18-TCS100 = 3; GD18-TCS1000 = 3; GD18-CO2 = 4; GD18-C = 4; PP7-CO5 = 3; PP7-TCS500 = 3; PP28-CO5 = 4; PP28-TCS100 = 4; PP28-TCS500 = 4; PND28-PM-CO5 = 2; PND28-PM-TCS100 = 2; PND28-PM-TCS500 = 2; PND28-PF-CO5 = 2; PND28-PF-TCS100 = 2; PND28-PF-TCS500 = 2. Animals were euthanized using CO_2_ and feces were collected from the colon of each animal. Fecal samples were collected from adult female rats to assess the impact of TCS during the pregnancy and postpartum (in female adult rats; GD6, GD18, PP7 and PP28) and to assess the impact of exposure to TCS in developing pups (pups at PND28). For samples collected on PP7, animals were placed in clean cages with no bedding.

### 2.3. Fecal Bacterial DNA and RNA Isolation

All fecal samples were collected aseptically from the colon of each animal into storage tubes and immediately transferred to liquid nitrogen except for those of dams on PP7 for which they were collected after placement into clean cages for collection. Upon freezing, samples were placed in dry ice for overnight shipment. Upon arrival at the National Center for Toxicological Research (NCTR, Jefferson, AR, USA), fecal bacterial DNA and RNA were isolated using Zymo ZR-duet DNA/RNA Miniprep (Zymo Research, Tustin, CA, USA). The flow-through was mixed with one volume of pure ethanol and transferred to a Zymo-Spin™ IIC Column. Both columns were washed with DNA/RNA wash buffer and eluted using DNase/RNase-free water. The quality and quantity of DNA and RNA were checked using a Cytation3 Cell Imaging Multimode Reader (BioTek, Winooski, VT, USA) and Qubit^TM^ Fluorometer (Thermo Fisher Scientific, Waltham, MA, USA).

### 2.4. 16s rRNA Next-Generation Sequencing

The bacterial DNA was assessed for quality using a Qubit^TM^ Fluorometer (Thermo Fisher Scientific) and diluted to 1 ng/µL. Equal amounts of DNA were used for the 16S rRNA sequencing using MiSeq. The complete methodology of sequencing has been previously described [40,41,42]. The microbial abundance analysis requires *n* = 3 but there were only *n* = 2 samples available in some experimental groups (e.g., PND). This lacuna was overcome by obtaining an additional value by averaging two values, with the results depicted as a heat map for an individual sample.

### 2.5. Sequencing of the Metatranscriptome and Examination of Orthogroups (KO)

For the metatranscriptome library construction, a Ribo-Zero Gold rRNA Removal Kit (Epidemiology) (Illumina, San Diego, CA, USA) was used (according to the manufacturer’s instructions) to deplete the rRNA in each sample. rRNA-depleted bacterial RNA was diluted to 5 ng/µL. An amount of 75 ng of RNA was used for library preparation using KAPA mRNA HyperPrep Kits (Kapa Biosystems) by following the manufacturer’s instructions. Following the library preparation, the final concentrations of all the libraries (Supplemental Table S1) were measured using a Qubit^®^ dsDNA HS Assay Kit (Life Technologies, Carlsbad, CA, USA), and the average library size was determined using an Agilent 2100 Bioanalyzer (Agilent Technologies, Santa Clara, CA, USA). The libraries were then pooled in equimolar ratios of 2 nM, and 8.5 pM of the library pool was clustered using cBot (Illumina) and sequenced paired end for 300 cycles using a HiSeq 2500 system (Illumina). The output data of transcriptomes were assembled using NGEN V16 using default settings. Read depth and coverage were retained and used in Meta Genome Rapid Annotation using Subsystem Technology (MG-RAST) to obtain expression data [43]. The advantage of using the MG-RAST portal for the analysis of metatranscriptomic data is that it offers default/automated parameters for quality control, annotation and comparative analysis.

### 2.6. Quantification of TCS in Rat Feces Using High-Performance Liquid Chromatography (HPLC)

The protocol of TCS detection and quantification using HPLC was adapted from Liu et al. with a minor modification [44]. An Agilent (Santa Clara, CA, USA) series 1200 LC was used and chromatography was carried out using a C18 column. The flow of the mobile phase was set at 1.2 mL min^−1^. The detection wavelength of DAD was 280 nm, and 10 μL sample extract or calibration standard solution were analyzed using isocratic elution using mixed acetonitrile and 0.01 mol L^−1^ acetic acid (pH 2.4; 60:40, *V/V*) as the HPLC mobile phase. The calibration curve was performed by preparing a stock solution of 1 mg/mL TCS and then diluting it to 0.1, 0.05 and 0.01 mg/mL. An aliquot of each standard was injected and the TCS elution profile monitored. The calibration curve was constructed by plotting the relative peak area versus the concentration of the TCS standard solution. Fecal samples from all animals were weighed and then diluted with methanol to 50 mg/mL. Samples were vortexed thoroughly and then centrifuged at 10,000× *g* for 10 min. The supernatant was filtered using a 0.22-micron nylon filter and 10 μL of each sample was injected directly into the HPLC system. Data collection and treatment were performed using an Agilent Chemstation.

### 2.7. Quantification of Bound and Unbound IgA in Rat Feces Using ELISA

Rat fecal samples were weighed and diluted to 50 mg/mL with phosphate buffer solution (PBS) containing PMSF (1 mM) and protease inhibitor cocktail solution (1 mM). Samples were vortexed rigorously and then centrifuged at 4 °C for 15 min at a speed of 900 g. The supernatant was collected and filtered through a 0.22-micron nylon filter. The filter was washed with 1 mL pure PBS and the flow-through collected to measure the unbound IgA level. To collect the bacteria-bound IgA, the same filter was washed with 0.05% Tween 20 and the flow-through was collected. A Rat IgA ELISA Kit (Bethyl Laboratories, Montgomery, TX, USA) containing a pre-coated 96-well strip plate was used to assess levels of IgA as described in the manufacturer’s protocol. Absorbance was measured on a Cytation 3 plate reader (BioTek) at 450 nm. The standard curve was fitted into a 4-parameter curve fitting equation which was programmed to calculate the analyte concentration in the original sample.

### 2.8. Statistics

The statistical analysis of intestinal commensal bacterial community structure due to exposure to TCS on specific phylum was calculated as the percent abundance as mentioned earlier [42]. Principal component analysis used data from Operational Taxonomic Units (OTUs) that represented the abundance levels of different phyla for PND28 samples only. Data were normalized using generalized logarithm transformation and then mean-centered and divided by the standard deviation of each variable. R software (Version 4) was used to generate the PCA plot.

All bar graphs are represented as mean values ± SE (standard errors). Statistical analysis was conducted using Statistical Package for the Social Sciences (SPSS^®^) software by performing repeated measure ANOVA for time-effect analysis and ANOVA with post-hoc analysis using the Tukey test for treatment differences. Statistical significance was considered when *p*-values were ≤0.05.

Since data from 16S analysis have shown less than 5% of standard deviation between biological replicates, the statistical analysis of metatranscriptomics data from random samples is considered significant if the differences are higher or lower than the 5% threshold.

## 3. Results

### 3.1. TCS Oral Exposure Is Associated with Alteration of Intestinal Commensal Bacterial Community Structure

This study aimed to evaluate potential changes in the intestinal bacterial community structure in dams upon TCS exposure during the gestational and lactational periods and in postnatal pups. For global analysis of the bacterial populations, 16S rRNA sequencing was performed and data were presented here at three different levels to show the taxonomic resolution: at the phylum level, at the genus level and at the species level.

**Figure 2:**
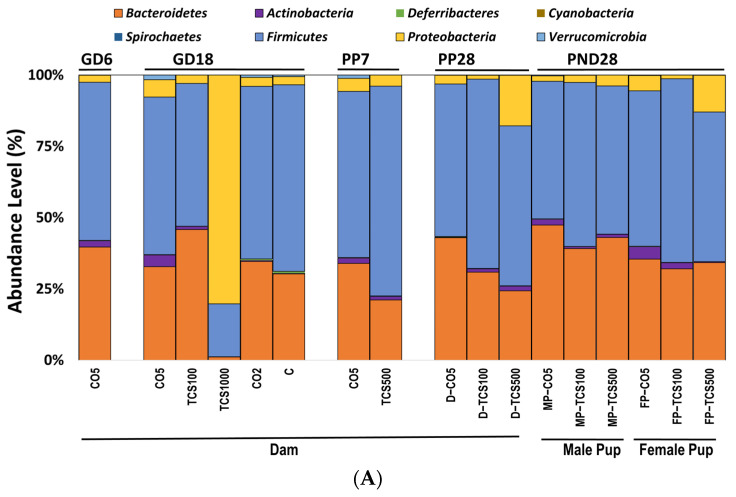

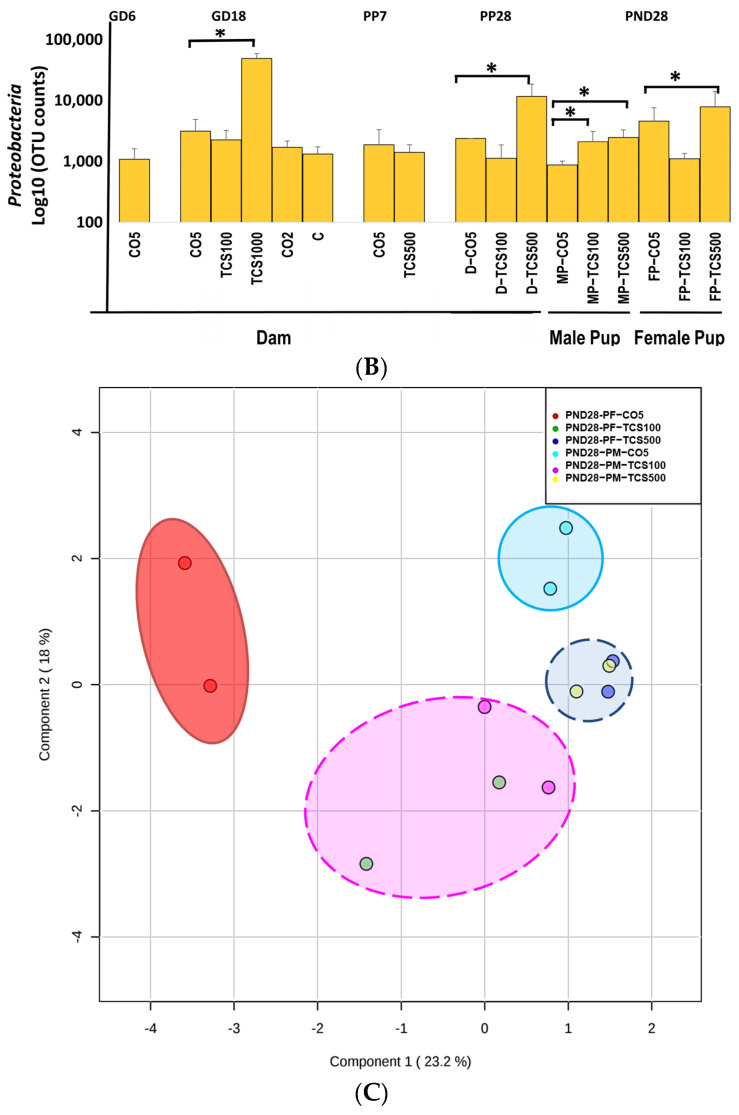
The analysis of intestinal bacterial composition at the phylum level. (**A**) The relative abundances of major bacterial phyla detected using the 16S rRNA sequencing method. (**B**) The number of OTUs of *Proteobacteria* in feces presented after Log10 transformation in dams (D) and pups (P). Error bars show standard error values. All abundance levels are expressed based on OTUs. Comparison is made within each experimental group (GD18, PP28 and PND28). *, *p* < 0.05 compared to control. (**C**) For the offspring, microbial assessments were conducted on PND28 pups exposed continuously via gestational, lactational and oral gavage daily from PND12 to 28 with TCS at two different concentrations (100 and 500 mg/kg/day) or control (corn oil 5 mL/kg/day). PCA depicting the differences between the microbiome of male pups (MP) and female pups (FP) before and after exposure to TCS and compared to corn oil control (CO5; 5 mL/kg corn oil). The solid outlines are control (CO5); dashed outlines are treatment groups [TCS100 (pink) or TCS500 (blue)].

At the phylum level, pregnant rats (GD18) exposed to TCS showed a dose-dependent response in bacterial abundance. At the lowest concentration (100 mg/kg/day), the abundance of *Bacteroidetes* increased in concert with the decrease in *Firmicutes* (Figure 2A).

In contrast, the highest concentration of TCS (1000 mg/kg/day) decreased both *Bacteroidetes* and *Firmicutes* at the phylum level, and at the same time, there was an increase in the pathogenic bacterial phylum *Proteobacteria* (Figure 2B). Daily exposure to TCS via oral gavage further changed the abundance of certain other phyla in adult rats (dams). At PP28, dams exposed to TCS (both concentrations of 100 and 500 mg/kg/day) showed lower levels of *Bacteroidetes* and increased abundance of *Proteobacteria* phyla (TCS 500 mg/kg/day) (Figure 2B). Moreover, rats exposed to 100 mg/kg/day TCS at PP28 showed a slight decrease in *Bacteroidetes* when compared to the same dose exposure at GD18.

At PP7, dams showed a perturbed intestinal microbiome, specifically, the abundance level of *Bacteroidetes* was comparatively lower during the TCS exposure (Figure 2A and Figure S1). Moreover, *Verrucomicrobia* phylum abundance was decreased both at PP7 and PP28 during the TCS exposure (Figure 2A). For the offspring, microbial assessments were conducted on PND28 pups exposed continuously via gestational, lactational and oral gavage daily from PND12 to 28 with TCS at two different concentrations (100 and 500 mg/kg/day) or control (corn oil 5 mL/kg/day). The control pups gavaged with corn oil only at 5 mL/kg/day showed gender differences in fecal microbiota (Figure 2A). Moreover, the phylum *Proteobacteria* was significantly increased during TCS exposure in adult rats. A similar finding was observed in both male and female TCS-exposed pups on PND28 (Figure 2B). In the offspring at PND28, total OTUs were higher in females as compared to males (Figure S2) and correlated with the level of TCS detected in feces (Figure S3). Furthermore, the microbial perturbation after TCS exposure in male pups was more pronounced in comparison to female pups and showed a significant increase in *Proteobacteria* at the lowest dose of TCS (100 mg/kg/day) (Figure 2B). The microbiota of male and female pups tended to respond similarly (increase in the phylum *Proteobacteria)* to the exposure of TCS even though the starting levels of phylum abundance were different (as shown in Figure 2A,B). Another important observation is that all groups of control dams (dams without corn oil (untreated) or orally gavaged with 2 mL/kg corn oil or 5 mL/kg corn oil) had the same level of *Proteobacteria.* A very similar result was observed in our previous studies [45]. Interestingly, the principal component analysis (PCA) unveiled that upon exposure to TCS, the microbial population in pups was related to the dose of TCS. This analysis used data from OTUs that represent the abundances of phyla for PND28 samples only. The *Firmicutes* phylum was the main driver of PC1 and the Bacteroidetes phylum was the main driver of PC2. Figure 2C shows that the microbiota of control male and female pups appear in different compartments. However, after TCS exposure, we found that fecal microbial populations appeared to aggregate at the same compartment depending on the dose used and there were no gender differences within each aggregate. Moreover, at the higher dose of TCS, males and females aggregated more tightly. The *Firmicutes* phylum was the main driver of PC1 and the *Bacteroidetes* phylum was the main driver of PC2.

**Figure 3:**
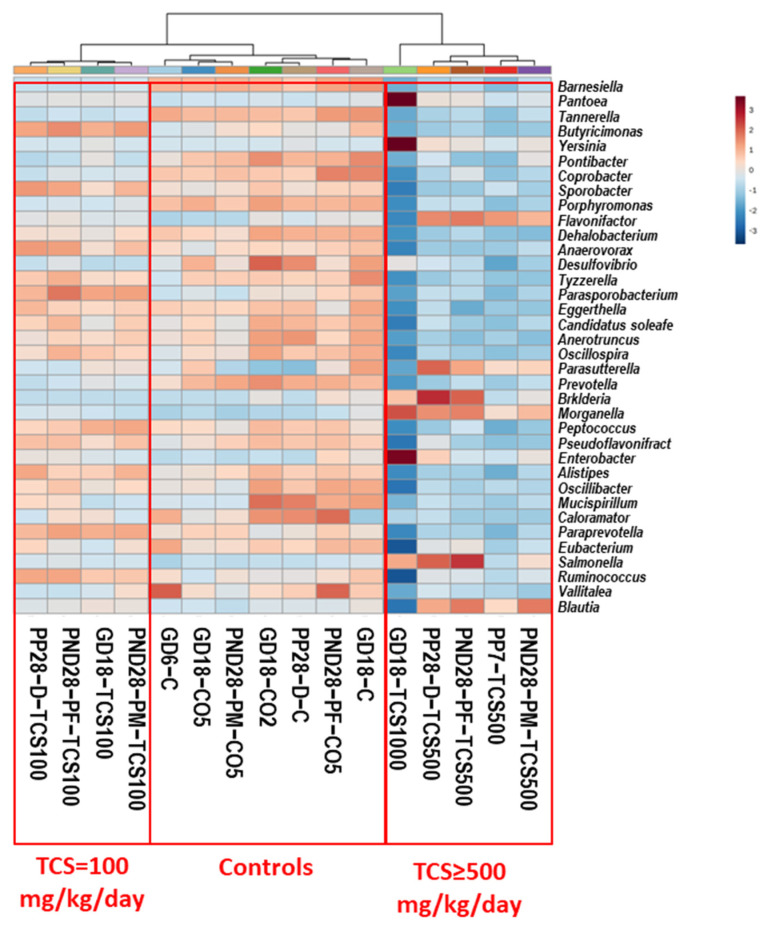
Heat map of the abundance of bacteria at the genus level based on OTUs. Each colored cell on the map corresponds to an OTU value, with genus in rows and samples in columns. Clustering was performed using Euclidean measurement. Only the top 36 genera that show significant ANOVA values are depicted here. Blue color means decrease in abundance and red color means increase in abundance. Heat map colors show the relative abundances of genera as shown in the scale at the right margin.

Analysis of 16S rRNA at the genus level showed that the microbiome response to TCS exposure was dose dependent. A total of 197 genera were identified in this analysis (Figure S4). The ANOVA analysis showed that 170 out of 197 genera were significantly affected by the tested treatment (*p* < 0.05). Figure 3 shows a heat map analysis of the top 36 genera with significant differences (ANOVA) across the different groups studied.

The cluster analysis showed three distinct groups: First, the control groups included all rats gavaged with corn oil (CO2 and CO5) only or not gavaged with oil (C; the untreated controls). Second, the group of rats treated with 100 mg/kg/day TCS was grouped independently of the rats’ age or sex to observe the global impact of TCS at the lowest level tested in this study. Third was the group of rats treated with 500 mg/kg/day or higher TCS. This clustering shows that the major genus abundance levels in feces decreased with TCS exposure in the 500 mg/kg/day treated rats. However, there were significant increases in several genera. We identified 11 genera that had increased abundance when compared to controls: *Erysipelatoclostridium*, *Lachnoclostridium*, *Anaerostipes*, *Parasutterella*, *Flavonifactor*, *Blautia*, *Enterobacter*, *Pantoea*, *Yersinia*, *Burkholderia* and *Morganella*. The comparison between dams and pups at PND28 showed different bacterial profiles after TCS gavage. However, out of 56 genera that increased in abundance, 20% were common between pups and dams, and of the 75 genera that decreased in abundance, 10% were similar between pups and dams. Of the 72 genera that showed no changes in abundance after TCS treatment, 46% were common between dams and pups. These results show that the exposure of pups to TCS via lactation may have led to the difference observed in the microbiota profiles between dams and pups.

The trend of abundance of genera in pups is different compared to that of the DAM (PP7). We organized all the genera that showed significant differences in abundance between TCS-treated and control rats into a table based on the levels of genus abundance. Figure S1 summarizes the list of genera that have either changed (increased or decreased) in pups (PND28) after exposure to TCS as compared to maternal (PP7) abundances. We identified a total of 20 genera that did not change after exposure to TCS upon exposure via lactation or oral gavage. Moreover, we identified 54 genera that showed decreases or increases in abundance in genera when compared to DAM (PP7).

**Figure 4:**
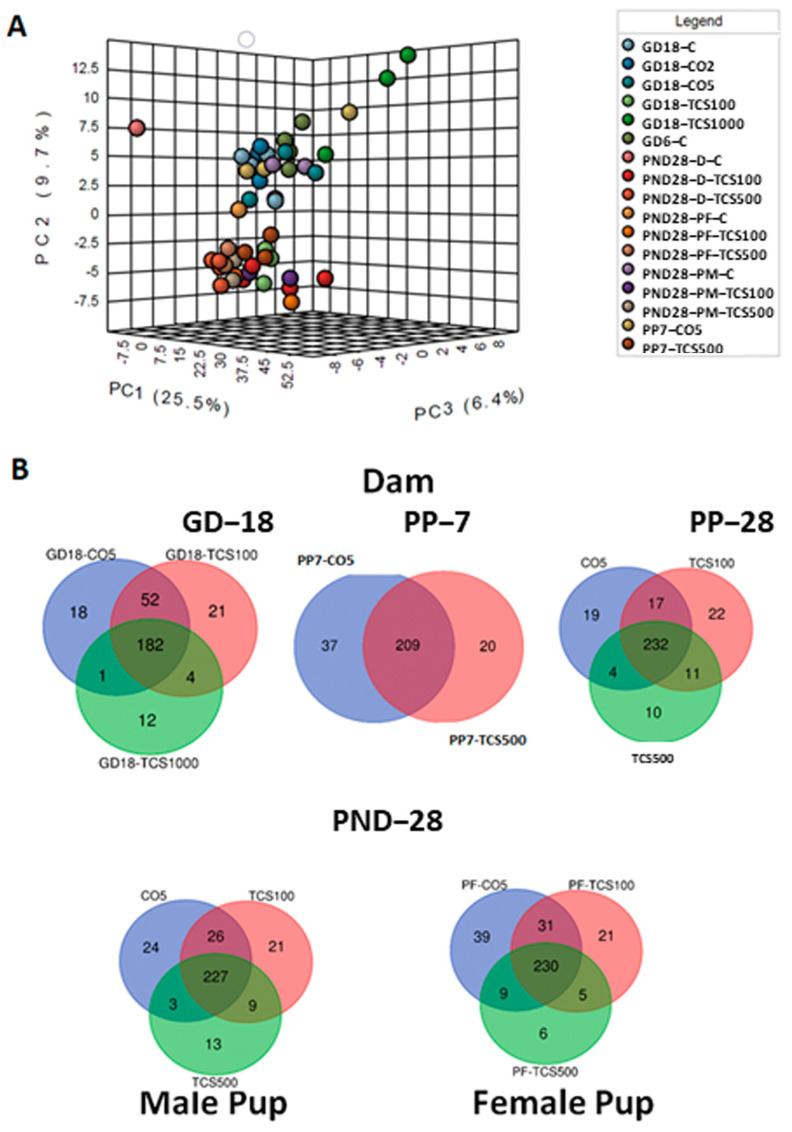
The analysis of bacterial composition at the species level. (**A**) Three-dimensional PCA plot based on OTU abundance levels. (**B**) Venn diagrams depicting numbers of common bacterial OTUs between different doses of TCS and controls in dams (top panel) and pups (lower panel).

Next, we examined the bacterial community only at the species level (Figure 4A). The PCA resulted in the segregation of animals based on the treatment and age variable. The distinction between both groups based on TCS treatment clearly shows that TCS perturbs the microbiome at different ages and sexes of rats and this effect is observed even at the lowest level of TCS used in this study (100 mg/kg/day). This observation led us to question whether the diversity and abundance of OTUs in TCS-treated and untreated rats were similar to their respective controls during the developmental stage. Venn diagrams (Figure 4B) elucidate the number of OTUs shared between the different groups studied. Some of the OTUs that were exclusively present in the TCS treatment group represented (but were not limited to) *Enterobacter hormaechei*, *Yersinia pseudotuberculosis*, *Streptococcus merionis*, *Ureibacillus suwonensis*, *Bacteroides oleiciplenus*, *Tyzzerella clostridium propionicum*, *Obesumbacterium* spp., *Enterococcus faecalis* and *Morganella psychrotolerans.* Out of these 37 unique OTUs, seven were also present in the pups at PND28 and these included: *Oxobacter* sp., *Caloramator* spp., *Methylobacterium komagatae*, *Menterobacter* spp., *Clostridium acetireducens*, *Anaerofustis stercorihominis* and *Lactobacillus* sp. Furthermore, the comparison between the microbiomes of dams and pups treated with TCS, independently of the dose used, showed the persistence of seven species that were absent in control samples including *Hespellia* spp., *Ruminiclostridium clostridium aldrichii*, *Planococcus maitriensis*, *Saccharofermentans acetigenes*, *Isoptericola* spp., *Escherichia coli* and *Yersinia pseudotuberculosis.*

The overall results of the 16S rRNA next-generation data show that TCS changed the composition of the bacterial community. To address if there was any correlation between the levels of TCS in the intestine and the abundances of different bacterial species, we quantified the amount of TCS present in feces.

### 3.2. The Relationship between TCS Excreted in Feces and the Number of OTUs

**Figure 5:**
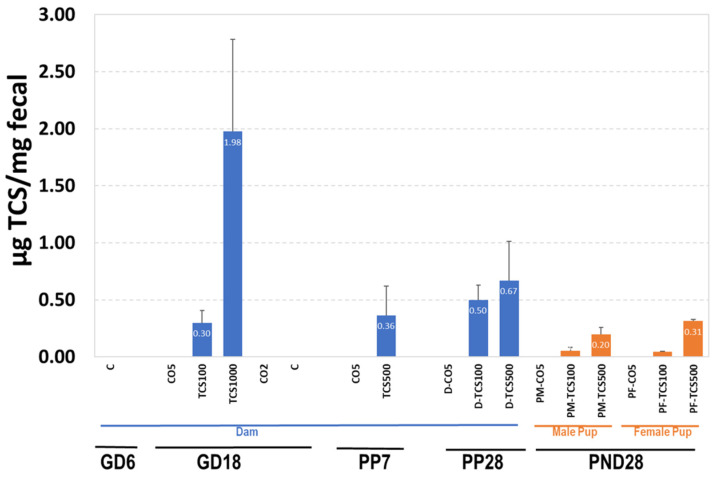
The quantification of TCS in feces using HPLC. The levels of TCS in dams and pups are expressed as μg TCS/mg feces.

Since TCS is partially eliminated via the fecal route, it is important to measure the levels of TCS in feces. The results of TCS expressed as μg TCS per mg feces are shown in Figure 5. TCS-treated pregnant adult rats (GD18) showed amounts ranging from 0.3 μg TCS/mg feces to 1.98 μg TCS/mg feces (Figure 5). At PP28, TCS levels in adult rats treated with 100 mg/kg/day increased by 67% from 0.3 to 0.5 μg/mg. This observed increase correlated with an increase in OTUs (Figure 5, Figures S2 and S3). The level of TCS in feces of rats treated with 500 mg/kg/day was higher (>0.50 μg TCS/mg feces) than levels in rats treated with 100 mg/kg/day (<0.50 μg TCS/mg feces). Even though the dose differences between groups was 5-fold, we did not observe a 5-fold increase in the amount of TCS in feces.

The pups exposed to TCS via gestation and lactation (with direct dosing for days 12–28 of lactation) showed measurable amounts of TCS in their feces that increased with dose. At PND28, the level of TCS detected in the feces of pups was lower compared to that of the dam (PP28) (Figure 5). The decreased excretion of TCS correlated with a decrease in OTUs detected using 16S rRNA sequencing, indicating persistent antibacterial activity of retained TCS (Figure 5, Figures S2 and S3). While comparing dams and their pups gavaged with the same dose of TCS at PND28, the adult rats showed a better capability to eliminate TCS via fecal excretion than pups (Figure 5).

The presence of TCS in the GI tract could have a direct effect on the fecal microbiota and can also affect the immune system. Given that IgA has been linked in shaping the microbiota composition and diversity [46], we wanted to determine if levels of IgA in feces allied with the changes in fecal microbiota after exposure to TCS.

### 3.3. The Unbound IgA Influences the Microbiome after TCS Exposure

**Figure 6:**
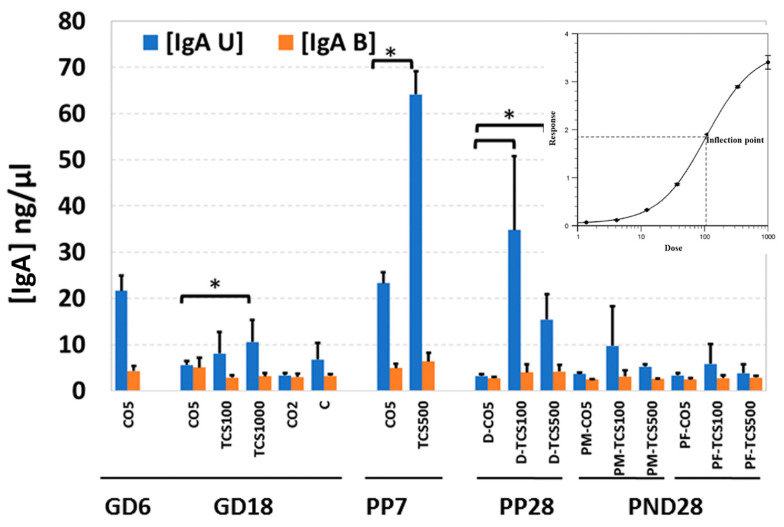
The quantification of IgA levels in maternal and offspring feces after exposure to TCS. The level of IgA in rat feces is expressed as ng per mg of feces. IgA U-secretory IgA (blue bars); IgA B = bacteria-bound IgA (orange bars). *, *p* < 0.05 compared to control. Error bars represent standard error values (*n* = 3/4).

Both secretory (fecal unbound) and bacteria-bound IgA were quantified in all stool samples. The levels of IgA in feces of control adult rats showed a significantly lower (*p* < 0.05) IgA level during pregnancy (GD6 to GD18) and until PP28 (Figure 6).

These results were completely opposite to the numbers of OTUs in the same fecal samples which increased between GD6 and PP28 (Figures S2 and S5). This is not unusual, because IgA levels are known to fluctuate during gestation and early developmental stages; once the microbiota reaches a balance, the levels of IgA decrease significantly [32]. TCS-gavaged rats showed a significant increase in levels of IgA in feces of rats treated with 1000 mg/kg/day TCS on GD18 (Figure 6). The dose of 1000 mg/kg was determined to cause behavioral changes and these dams were removed from the study by PP7. At PP28, dam groups treated with TCS at 100 mg/kg/day and 500 mg/kg/day showed significantly higher IgA levels compared to controls. This observed increase, between GD18 and PP28, correlated with an increase in OTUs in the same fecal samples (Figure 6, Figures S2 and S4).

In PND 28 pups, the levels of IgA were comparable between treated and control pups. No sex differences were observed between the levels of IgA detected in the feces of rat pups treated with TCS. The levels of bacteria-bound IgA were very close to the limit of detection of the ELISA kit. For this reason, the results did not show significant differences between the different treatments (Figure 6). More sensitive detection techniques will be required to ascertain if there is a correlation between levels of bound IgA with changes in the microbiome due to TCS exposure.

Taken together, all these results suggest a significant role of different bacterial genera in promoting a differential response based on the age and pregnancy/lactational status of the animal and the doses of TCS used. Here, it remains important to understand the functional content of intestinal bacteria in order to link it to the dynamics of bacterial changes in the gut.

### 3.4. Examination of Intestinal Bacterial Functional Content

**Figure 7:**
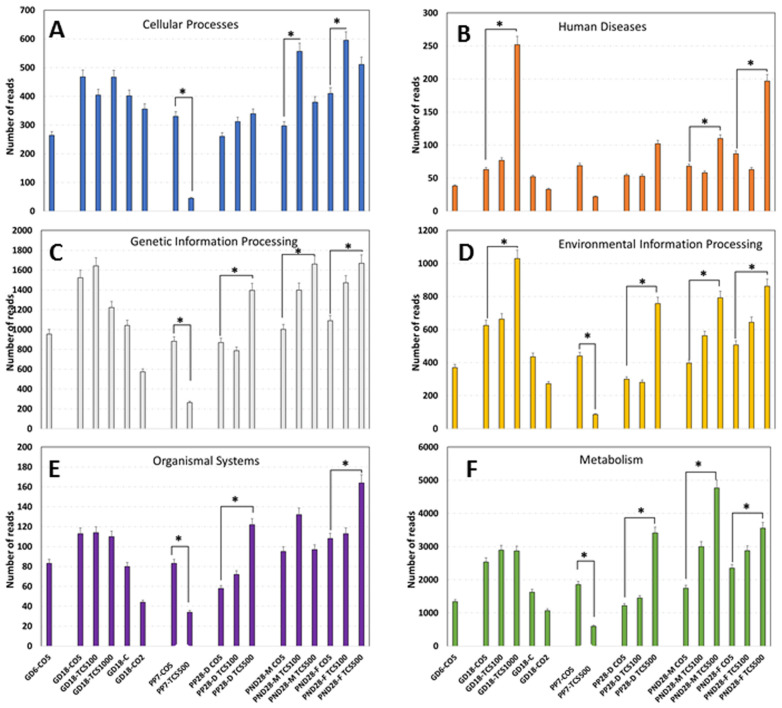
Functional gene expression abundances of KEGG Level 2 pathways in the gut microbiome. Abundances of functional categories of intestinal bacteria as measured using the metatranscriptomic analysis are provided as the numbers of reads in each experimental group encoding (**A**) cellular processes, (**B**) human diseases, (**C**) genetic information processing, (**D**) environmental information processing, (**E**) organismal system and (**F**) metabolism depicted in the graph. *, *p* < 0.05 in comparison to corresponding control.

The analysis of functions encoded in the genomes of intestinal bacteria revealed six different categories of orthogroups (KO) including cellular processes, human diseases, genetic information processing, environmental information processing, organismal systems and metabolism (Figure 7). Notably, the metabolism function was the most abundant function among all six categories with numbers of reads ranging from 497 to 4765. At GD18, the metabolism function reads in rats exposed to TCS were not different from their corresponding control; however, as the duration of TCS exposure increased, there were significantly lower reads of the metabolism category in dams exposed to TCS at PP7. However, at PP28, dams showed a significant increase in the number of reads in this category. At PND28, the number of reads in TCS-exposed pups increased in a dose-dependent manner. In relation to the levels of TCS detected using HPLC in feces, we can clearly see a pattern—the higher the exposure dose of TCS, the higher the amount of TCS in the feces. Moreover, at PP7, the lower values of metabolism reads for dams gavaged with TCS were in correlation with the high levels of TCS detected in the feces of the same animals (Figure 5). Besides the metabolism category, two other orthogroups stand out by the early increase in gene expression at GD18 (Figure 7B,D). In fact, at GD18, we found that rats gavaged with TCS at 1000 mg/mL/day showed a significant increase in reads in relation to both categories.

The sex comparison of fecal metatranscriptomics data between pups at PND28 revealed that female pups had higher numbers of human diseases-related reads in comparison to male pups at the highest dose of triclosan used. Similarly, the number of reads for organismal systems-related genes doubled in female pups compared to male pups at PND28 after gavage with the highest dose. None the less, at the metabolism level, male pups had a 34% higher number of reads compared to female pups at PND28. Data from 16S rRNA sequencing have suggested that the sex of rats has a lower impact on microbial composition changes (Figure 2). However, functional analysis of genes expressed using microbiome data clearly shows that the sex of the host can have a clear impact on the function of bacterial communities (Figure 7) and this is at an age where adult levels of sex hormones have not yet begun to be secreted.

## 4. Discussion

Humans are exposed daily to a broad spectrum of xenobiotics in different forms and doses [6,47,48]. Concerns about the voluntary or involuntary ingestion of these chemicals continues to rise due to potential adverse health outcomes. Specifically, there is growing evidence that the perturbation of the microbiome is linked to issues such as body weight changes [49,50,51] and insulin resistance or altered hormonal levels [52,53]. Moreover, the intestinal microbiota is also known to influence gut development [54]. A better understanding of the outcomes of xenobiotic exposure is necessary and should consider diverse variables such as exposure, pregnancy status, age and sex [55]. Although humans are exposed to low doses of many chemicals, their presence in surface, ground and drinking water indicates their persistence and potential accumulation in the human body at high doses [6,55,56]. TCS, previously a commonly used antimicrobial compound, has raised public concerns due to evidence that suggests its link to alteration of host physiology [51], disruption of microbial communities [9,57] and an increase in bacterial resistance [7,58,59,60]. Moreover, TCS is also linked to induce risk among neonates via breastmilk intake [5,56,61]. The present study was designed to determine the effect of TCS on the gastrointestinal microbiome during pregnancy, as well as impact on the offspring exposed indirectly in utero and directly dosed for part of the lactational period. Moreover, this study is designed to assess the sex-dependent impacts in male and female pups. The results from this study demonstrate that early exposure to TCS can have a sex-dependent impact on the structure of the bacterial community in the gut.

The doses of TCS used in this study ranged from 100 to 1000 mg/kg/day, which correspond to the range of 15–150 mM TCS solution daily. In a human study by Lin et al., it was found that mouth rinse with 15 mL solution containing 0.03% (1 mM) TCS resulted in the retention of 0.26–0.33 μM TCS in plasma [62]. Moreover, the lower dose (100 mg/kg/day) of TCS is NOAEL for developmental toxicity [38]. The two higher doses (500 and 1000 mg/kg/day) selected in this study provide a threshold approach for dose response to xenobiotics [39].

In our study, significant changes in the microbiome structures in rats were noted at all doses of TCS used. The increase in *Proteobacteria* in all TCS-treated rats could be a good indication of dispersion of the bacterial communities. These observation is consistent with a recent study that shows TCS exposure in mice increases *Deltaproteobacteria* in the intestine [63]. The enrichment of the *Proteobacteria* phylum has been proposed as a diagnostic signature in numerous diseases such as diabetes, colitis and malnutrition [64]. Thus, the evaluation of such dysbiosis could serve as a predictive toxicity of a xenobiotic. Other noted genera including *Escherichia*, *Morganella*, *Turicibacter*, *Enterobacter*, *Salmonella*, *Enterococcus* and *Clostridium*, known to comprise many pathogenic bacteria, were also significantly increased after exposure to TCS. In contrast, certain genera were found to be completely or near completely abolished after exposure to high doses of TCS including *Butyricicoccus*, *Candidatus Azobacteroides*, *Cryptobacterium*, *Tannerella*, *Neisseria* and *Flavobacterium.* Many clinical studies have shown that certain genera such as *Butyricicocus* are important for maintaining a healthy gut and preventing inflammatory bowel disease [65]. These findings suggest that exposure to TCS could have long-term effects on the microbiome and may possibly contribute to serious inflammatory diseases. The intestinal microbiome, however, uses certain mechanisms to oppose the negative impact of xenobiotics. Interestingly, the *Lactobacillus* genera was shown to increase after exposure to TCS during all stages of the study. This finding is in with agreement with that of Zang et al. who showed that *Lactobacillus plantarum ST-III* has been found to increase the diversity of the gut microbiota in zebrafish, thereby reducing the toxicity of chronic exposure to triclosan [66].

The daily exposure of adults and pups to TCS in the current study reflects the regular use of TCS-containing consumer products. The data indicate that early-life exposure could be detrimental. For example, the perinatal exposure of pups to TCS via lactation resulted in changes in the microbiome. Our study detected 54 intestinal bacterial genera that were altered (decreased or increased) after TCS exposure during PP7 and showed similar patterns in pups at PND28 (Figure S1). We also found that at PND28, out of all the genera that significantly decreased due to TCS oral exposure, 76% of them were specifically decreased in pups that were exposed to TCS during pregnancy. The effects of postnatal exposure to TCS on the microbiome have been discussed in many studies [2,25,51,56]. A toxicology study of TCS determined that adolescent rat microbiota were more vulnerable to chemical perturbation than the adult microbiome [28]. Even though previous research showed that oral gavage of pups with TCS could perturb the microbiome, those studies failed to mimic the transfer of microbes during early development.

**Figure 8:**
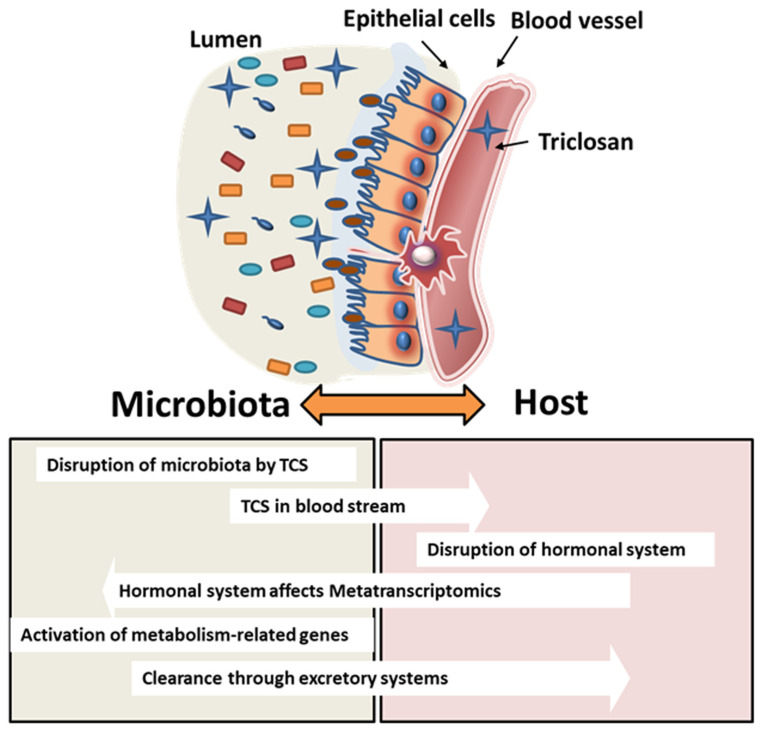
Schematic figure depicting the interaction between the microbiota and the host. TCS exposure may disrupt the microbial colonization on the intestinal mucosa. This may lead to intestinal epithelial cell–cell junction disruption that may lead to increases in TCS absorbed in the blood stream impacting further downstream processes.

Pharmacokinetic analysis of TCS after oral exposure shows that the half-life of TCS is 21 h [67]. Urinary half-life excretion, on the other hand, is known to be 11 h and constitutes 57% of the total excretion. The remaining TCS is either excreted via feces or biodistributed from the blood to tissues [62]. In our study, the elimination of TCS via feces showed differences between adult pregnant/lactating rats and pups. Starting on GD6, dams were dosed with TCS daily, and starting at PND12, both adults and pups were dosed via oral gavage with identical doses of TCS; the analysis revealed that adult rats had higher levels of TCS secretion in feces compared to PND28 pups. Based on metatranscriptomics data analysis of our study, we identified that the metabolism genes in bacteria exposed to TCS (in both dams and pups) increased significantly in comparison to the control, which implied that bacteria may play an important role in the clearance of TCS from the host. This finding is supported by the fact that the increase in TCS elimination via its conjugates is achieved over time with the increase in bacterial metabolism capability. In our results, male pups showed higher metabolism-related genes compared to female pups when exposed to the highest dose of TCS. This finding is in agreement with the HPLC analysis data that show that males had a low amount of TCS in feces compared to females. While it is still not clear whether sex can play a role in TCS metabolism, a survey from the National Health and Nutrition Examination Survey (NHANES) showed that males had higher urinary TCS than females [68]. On the other hand, metatranscriptomics data have shown a higher increase in human disease-related genes and organismal system genes in female pups. The sex differences found in the metatranscriptomics data were different from our findings on the microbiome analysis data. In other words, the bacterial communities in both male and female pups are similar, however, their gene expression is different. This finding can be explained by the impact of the host on the microbiome. TCS can be absorbed into the bloodstream after being orally gavaged and can affect the endocrine system in both males and females. The effect of TCS on the hormonal system [4,48,50,51], as well as glucose tolerance [69], can influence indirectly bacterial communities not by changing their composition but rather by using the same communities to clear a xenobiotic from the host system. Schematic Figure 8 details the interaction between the bacterial communities and the host after TCS gavage in rats.

Moreover, adult rats that were directly exposed to 5-fold higher doses of TCS did not show a 5-fold increase in fecal excretion. Adult rats treated with 500 mg/kg/day had a 35% increase in fecal excretion compared to rats treated with 100 mg/kg/day. These results show that as the TCS dose increases, the host mechanism of elimination of the chemical may not be as efficient. Further xenobiotic metabolism data and metabolic activity studies could elucidate how TCS is eliminated.

The observed changes in abundance of different bacterial species in the feces of rats exposed to TCS may be influenced by different factors. First, it is known that TCS can be bactericidal or bacteriostatic based on concentration [70]. Second, the host immune system could play a major role in orchestrating the response to TCS exposure via different mechanisms [54]. In order to maintain intestinal homeostasis, the mucosal immune system selectively recognizes and responds to pathogenic species. IgA is the predominant antibody isotype produced at mucosal surfaces and is a critical mediator of intestinal immunity [71,72]. The correlation (OTU:TCS and OTU:IgA) used in this study is a cross-correlation since we are comparing both variables at different time points. The cross-correlation analysis takes into consideration the time points at which the data were taken, which allow us to understand if the changes in the variables studied are time-dependent. The correlation between the results of IgA levels in feces and OTUs could be an indication of the involvement of the mucosal immune system in reducing inflammation that could have been caused by the emergence of pathogenic bacteria such as *Proteobacteria* in all TCS-treated rats. The increase in IgA production in feces could be a good indication of dysbiosis. However, it remains unclear whether the reduction in IgA is indicative of clearance of infection/toxicity or the failure of the host to defeat TCS-related toxicity. There is no doubt that the host–xenobiotic–microbiome is resilient and can adapt to many changes; however, further xenobiotic metabolism studies could help us understand the host response to TCS exposure and correlate the finding with the results obtained in this investigation.

## 5. Conclusions

This study shows that early exposure to TCS can impact the microbiome of rats, including changes in the microbial diversity. In conclusion, this study reveals that a higher concentration of TCS decreases the abundance of *Bacteroidetes* and *Firmicutes* at the phylum level; at the same time, there was an increase in *Proteobacteria*, a phylum with several representative pathogenic bacterial species. The increased abundance of *Proteobacteria* in all TCS-treated rats along with increased abundance of OTUs that belong to pathogenic bacterial communities could serve as a signature of TCS-induced dysbiosis. Moreover, the developmental exposure to TCS in pups had a sex-dependent impact on the structure of the bacterial community in the gut. Finally, the correlation between the results of IgA levels in feces and OTUs could be an indication of the involvement of the mucosal immune system for reducing the inflammation caused by pathogenic bacteria. These findings enhance our understanding of the impact of environmental toxicants on the composition of the gut microbiome and, potentially, on human health. Collectively, our results address the knowledge gap in the growing concerns related to the exposure of xenobiotics (such as TCS) and risk assessment of the gastrointestinal tract.

## Data Availability

The datasets of the study will be available as per the guidelines of the U.S. Food and Drug Administration data sharing policy.

## Supplementary Materials

The following supporting information can be downloaded at: https://www.mdpi.com/article/10.3390/jox14010012/s1, Table S1: Concentration of total RNA, final library concentration and average library size; Figure S1: The list of genera with abundance levels in pups (PND28) after exposure to TCS as compared to maternal (PP7). Several intestinal bacterial genera that were altered (decreased or increased) after TCS exposure at PP7. Perinatal exposure of pups to TCS resulted in changes in the microbiome pups at PND28; however, there was difference in the abundance of genera in dam and pups; Figure S2: Total OTUs detected using next generation sequencing platform (16S) of rat intestinal microbiota. The decrease in OTUs detected at PP7 correlated with the decrease excretion of TCS as shown is Figure 5; Figure S3: Correlation graphs between total number of OTUs and the level of TCS detected in feces. (a) Total OTUs and TCS in adult control animals; (b) Total OTUs and TCS in adult animals exposed to 100 mg/kg/day TCS; (c) Total OTUs and TCS in dam (PP7) and male pups (PND28) exposed to 500 mg/kg/day TCS; (d) Total OTUs and TCS in dam (PP7) and female pups (PND28) exposed to 500 mg/kg/day TCS; Figure S4: Heat map of the abundance of bacteria at the genus based on OTUs. Each colored cell on the map corresponds to an OTU value, with genus in rows and samples in columns. Clustering was made with Euclidean measurement. Blue color means decrease of abundance and red color means increase of abundance. Heat map color shows the relative abundance of genus as shown in the scale at the right margin; Figure S5: Correlation graphs between total number of OTUs and the level of IgA detected in feces. (a) Total OTUs and IgA (unbound) in adult control animals; (b) Total OTUs and IgA (unbound) in adult animals exposed to 100 mg/kg/day TCS; (c) Total OTUs and IgA (unbound) in dam (PP7) and male pups (PND28) exposed to 500 mg/kg/day TCS; (d) Total OTUs and IgA (unbound) in dam (PP7) and female pups (PND28) exposed to 500 mg/kg/day TCS.

## List of Abbreviations

EPA	Environmental Protection Agency
FDA	Food and Drug Administration
GD	Gestation day
IgA	Immunoglobulin A
NOAEL	No-observed adverse effect level
OTU	Operational taxonomic unit
PCA	Principal component analysis
PND	Postnatal day
PP	Postpartum
TCS	Triclosan
