# Supplementary material for: Early Developmental Exposure to Triclosan Impacts Fecal Microbial Populations, IgA and Functional Activities of the Rat Microbiome
Source: J Xenobiot. 2024 Feb 1;14(1):193–213. doi: 10.3390/jox14010012 (PMC10885032; doi:10.3390/jox14010012)
Supplement: Supplementary file 1 [file jox-14-00012-s001.zip › jox-2773854-supplementary.pdf]

Supplementary data

Early Developmental Exposure to Triclosan Impacts Fecal Microbial Populations, IgA and Functional Activities of the Rat Microbiome

Mohamed Lahiani [1], Kuppan Gokulan [1], Vicki Sutherland [2], Helen C. Cunny [2], Carl E. Cerniglia [1,†] and Sangeeta Khare [1,*]

[1] Division of Microbiology, National Center for Toxicological Research, U.S. Food and Drug Administration, Jefferson, AR 72079, USA; hassen86us@gmail.com (M.L.); kuppan.gokulan@fda.hhs.gov (K.G.)

[2] National Institute of Environmental Health Sciences, National Institutes of Health, Durham, NC 27709, USA; vicki.sutherland@nih.gov (V.S.); cunnyh@niehs.nih.gov (H.C.C.)

[*] Correspondence: sangeeta.khare@fda.hhs.gov; Tel.: +1-870-543-7519

[†] This research article is dedicated to Carl E. Cerniglia, who suddenly passed away during the preparation of this manuscript.

Table S1. Concentration of total RNA, final library concentration and average library size.

Sample	RNA Concentration (ng/uL)	Library Concentration (ng/uL)	Avg Library size (bp)
**GD6-C**	15.10	44.00	518
**GD18-CO5**	15.00	46.80	528
**GD18-TCS100**	14.40	49.20	558
**GD18-TCS1000**	13.60	49.20	494
**GD18-CO2**	14.50	52.20	548
**GD18-C**	16.20	42.60	524
**PP7-CO5**	14.60	46.20	513
**PP7-TCS500**	7.30	12.70	275
**PND28-M CO5**	14.90	47.60	544
**PND28-F CO5**	15.40	44.20	533
**PP28-D-CO5**	14.00	45.60	539
**PP28-D-TCS100**	14.60	45.00	491
**PND28-M-TCS100**	14.50	47.20	542
**PND28-F-TCS100**	14.40	47.40	470
**PND28-D-TCS500**	16.30	49.60	485
**PND28-F-TCS500**	15.10	49.60	456
**PND28-M-TCS500**	14.00	50.00	484

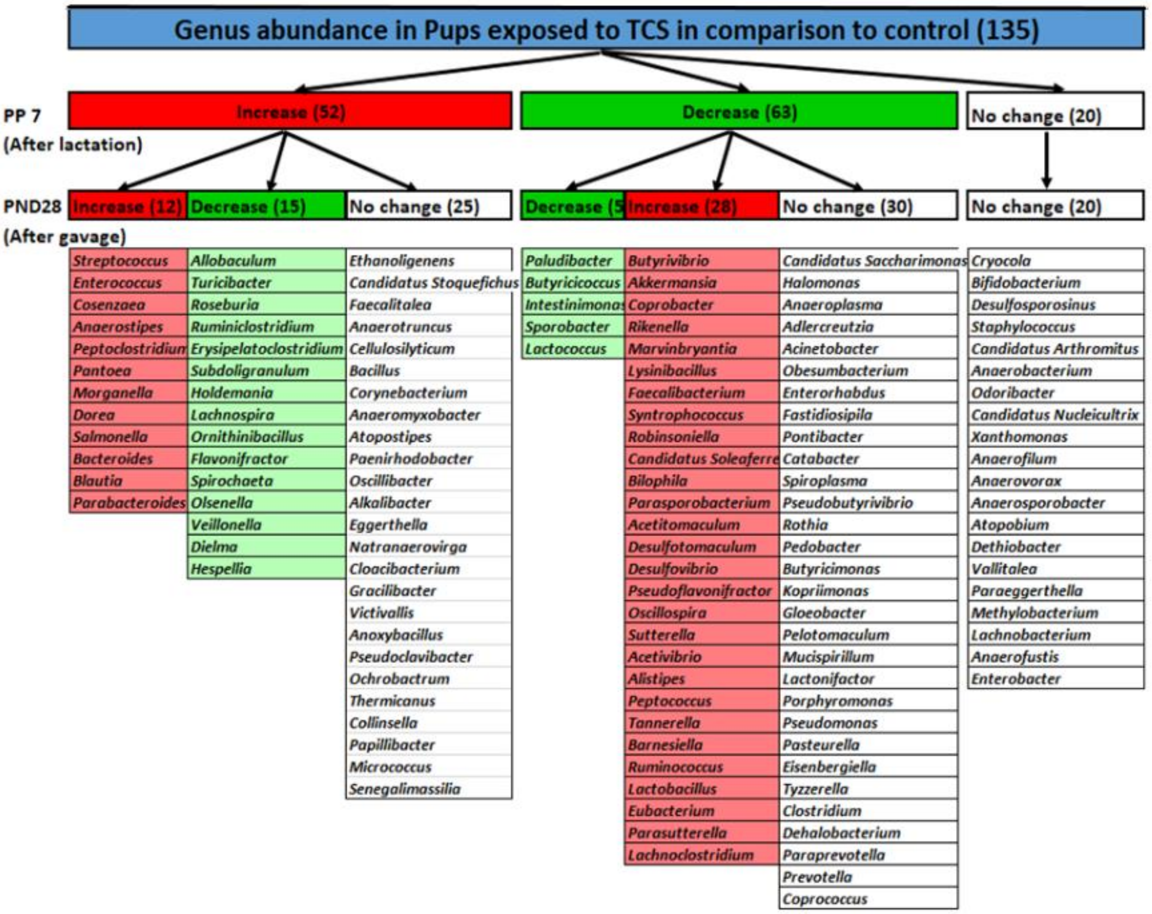

Figure S1. The list of genera with abundance levels in pups (PND28) after exposure to TCS as compared to maternal (PP7). Several intestinal bacterial genera that were altered (decreased or increased) after TCS exposure at PP7. Perinatal exposure of pups to TCS resulted in changes in the microbiome pups at PND28; however, there was difference in the abundance of genera in dam and pups.

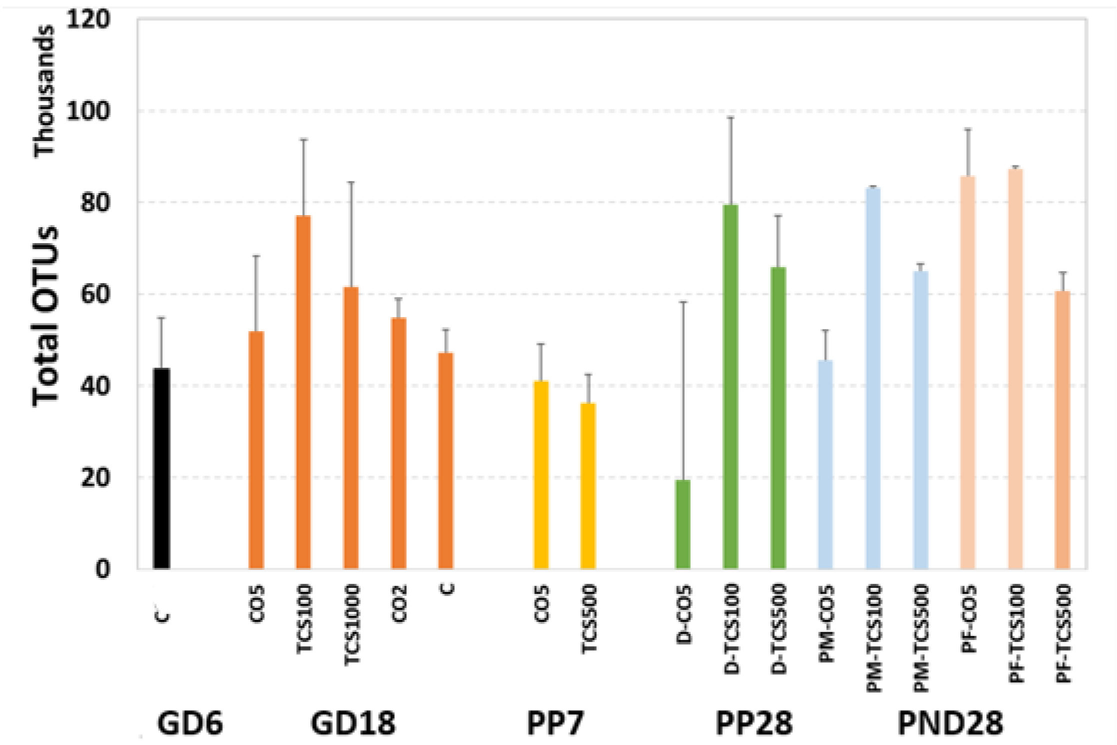

Figure S2. Total OTUs detected using next generation sequencing platform (16S) of rat intestinal microbiota. The decrease in OTUs detected at PP7 correlated with the decrease excretion of TCS as shown in Figure 5.

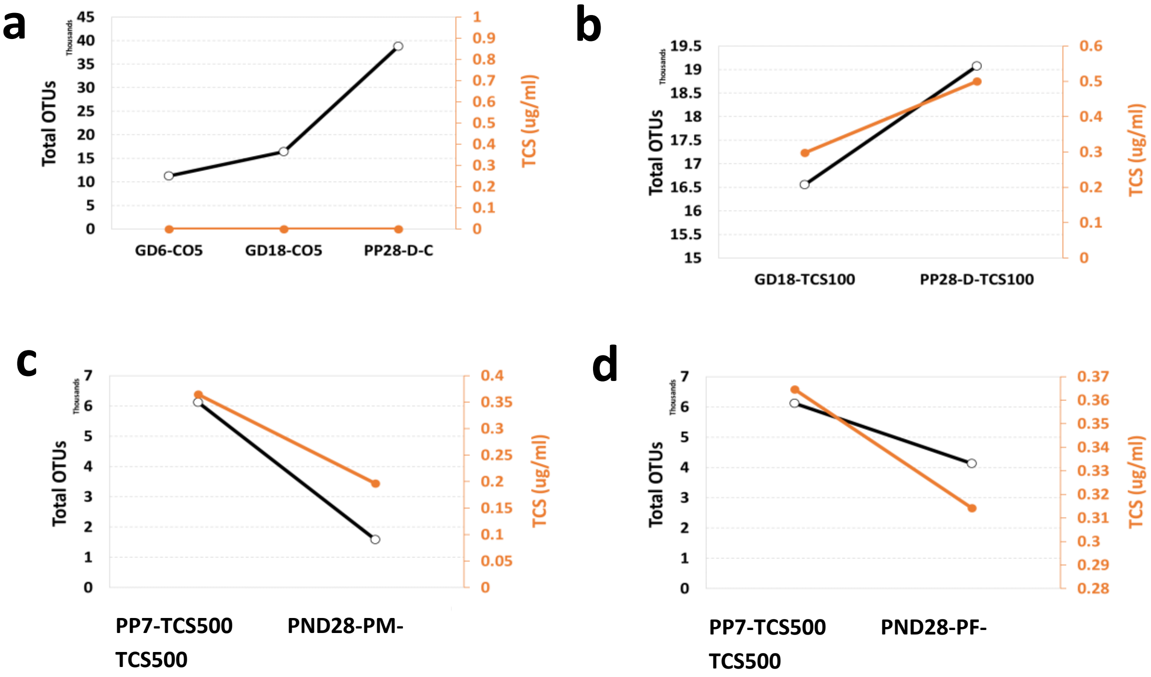

Figure S3. Correlation graphs between total number of OTUs and the level of TCS detected in feces. a) Total OTUs and TCS in adult control animals; b) Total OTUs and TCS in adult animals exposed to 100 mg/kg/day TCS; c) Total OTUs and TCS in dam (PP7) and male pups (PND28) exposed to 500 mg/kg/day TCS; d) Total OTUs and TCS in dam (PP7) and female pups (PND28) exposed to 500 mg/kg/day TCS.

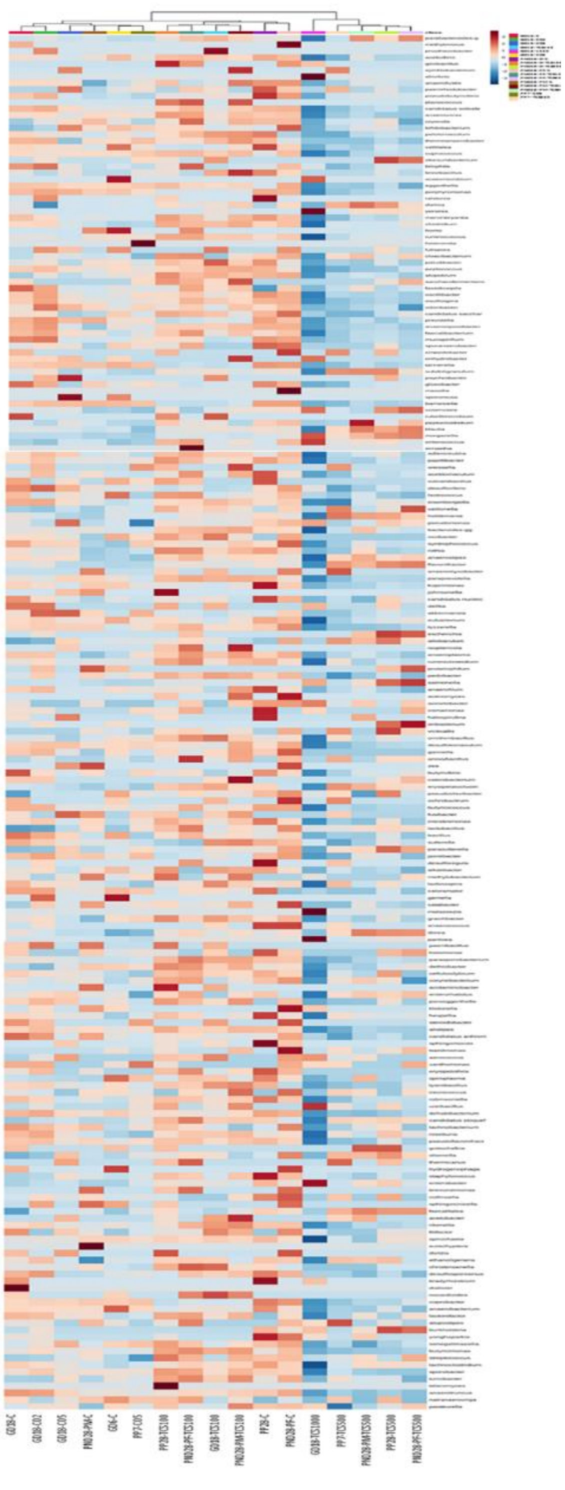

Figure S4. Heat map of the abundance of bacteria at the genus based on OTUs. Each colored cell on the map corresponds to an OTU value, with genus in rows and samples in columns. Clustering was made with Euclidean measurement. Blue color means decrease of abundance and red color means increase of abundance. Heat map color shows the relative abundance of genus as shown in the scale at the right margin.

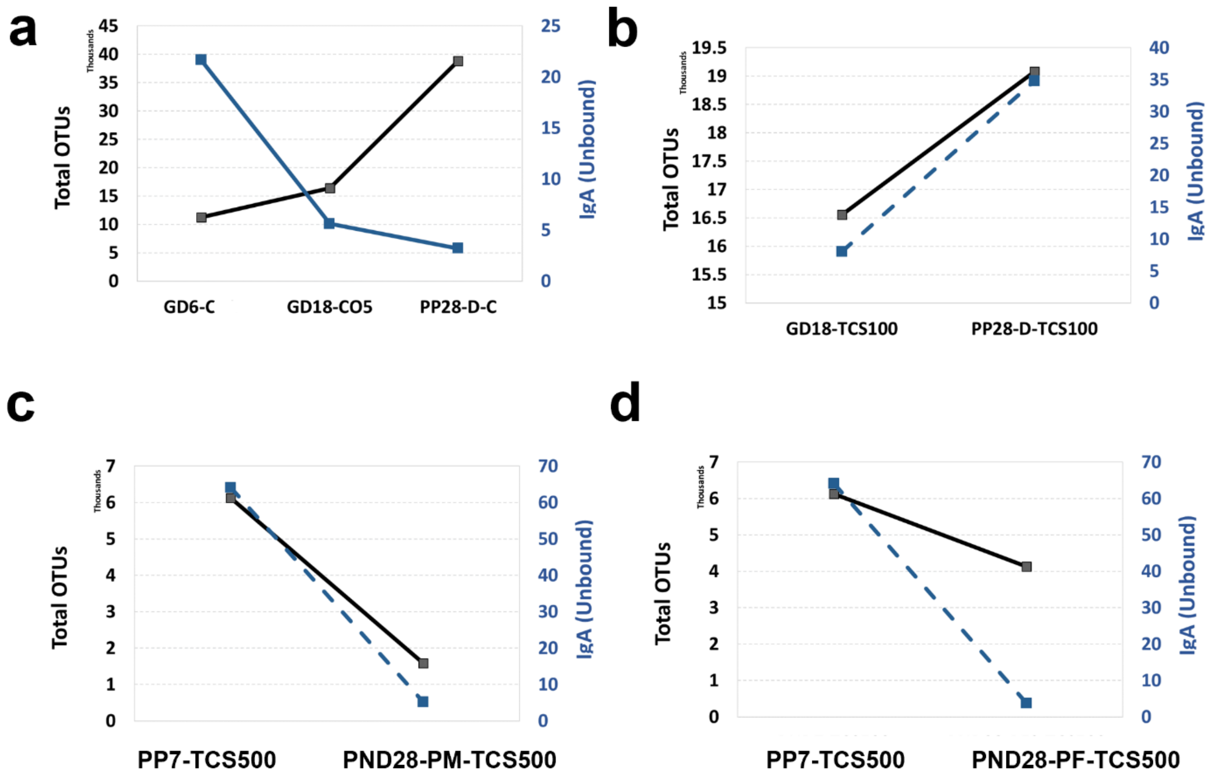

Figure S5. Correlation graphs between total number of OTUs and the level of IgA detected in feces. a) Total OTUs and IgA (unbound) in adult control animals; b) Total OTUs and IgA (unbound) in adult animals exposed to 100 mg/kg/day TCS; c) Total OTUs and IgA (unbound) in dam (PP7) and male pups (PND28) exposed to 500 mg/kg/day TCS; d) Total OTUs and IgA (unbound) in dam (PP7) and female pups (PND28) exposed to 500 mg/kg/day TCS.
